# Identification of a New Prediction Model for Bladder Cancer Related to Immune Functions and Chemotherapy Using Gene Sets of Biological Processes

**DOI:** 10.1155/2022/4740686

**Published:** 2022-10-30

**Authors:** Huidong Zhou, Guo Huang, Tao Chen, Jianglei Zhang, Jun Ouyang

**Affiliations:** ^1^Department of Urology, The First Affiliated Hospital of Soochow University, Suzhou, Jiangsu, China; ^2^Department of Urology, The Fourth Hospital of Changsha, Changsha, Hunan, China; ^3^Hengyang Medical College, University of South China, Hengyang, Hunan, China; ^4^Hunan Province Key Laboratory of Tumor Cellular & Molecular Pathology, Cancer Research Institute, Hengyang Medical School, University of South China, Hengyang, Hunan, China

## Abstract

**Background:**

Biological processes serve crucial functions in the initiation and development of cancer. Therefore, we constructed and validated a model for bladder cancer (BLCA) with good predictive power for immunity, prognosis, and therapy.

**Methods:**

Using the expression of the gene sets based on biological processes, BLCA patients were divided into three clusters by consensus cluster analysis. By performing LASSO regression analysis twice, key genes were selected, and the biological processes-related genes' (BPRG) score was calculated. Differences in immune infiltration, tumor microenvironment, tumor mutation burden, immunotherapy, and sensitivity towards chemotherapy were analyzed between two groups divided by BPRG score.

**Results:**

Good accuracy was observed for the three clusters. They showed different prognoses and levels of immune cell infiltration. The selected key genes were mainly enriched in immune-related pathways. The high-BPRG score group was related to poor prognosis, higher immune cell infiltration, interstitial scores, and increased tumor mutation. Moreover, the effects of immunotherapy were good, and those of chemotherapy were poor.

**Conclusion:**

Overall, key genes may be involved in various complex immune regulation processes. Therefore, the quantification and verification of the BPRG score are expected to facilitate the understanding of the immunosuppressive microenvironment in BLCA and guide the choice of chemotherapeutic drugs and immunotherapeutic regimens and help predict the prognoses of patients with BLCA.

## 1. Introduction

Bladder cancer (BLCA) is a common malignancy worldwide. The Centers for Disease Control and Prevention in the United States estimated 83,730 new cases of BLCA in 2021 [1]. Once the tumor progresses to the local advanced or the metastasis stage, the effects of traditional treatment methods, including surgery and chemotherapy, are not favorable [2]. Immune checkpoint inhibitor (ICI) therapy, such as CTL-associated protein 4 (CTLA4), programmed death-ligand 1 (PD-L1), and programmed death 1 (PD1), has brought new hope for the treatment of advanced BLCA [3]. Although BLCA is immunogenic cancer with characteristics of high tumor mutation burden (TMB) and heterogeneity [4], the efficacy of ICI treatment in many patients is insignificant. Several factors including the tumor microenvironment (TME) influence the effectiveness of ICI treatment. Coupled with high recurrence rates and risk of metastases in BLCA, only a few biomarkers can well predict these patients' prognosis and therapeutic effectiveness [5]. Therefore, the identification and quantification of molecular maps that affect the choice of drug treatment regimens are crucial. With recent advancements in the understanding of the biological behavior of BLCA and the progress in high throughput sequencing technology, the identification of new indicators to predict the prognosis of patients with BLCA is possible [6].

Many biological processes (BP) are linked to and altered during the initiation and development of cancer [7]. Therefore, determining BPs involved and quantifying the expressions of relevant genes in the study of cancer cell behavior are crucial. In addition, linking treatment sensitivity of some key components of BPs may also facilitate personalized medication guidance [8]. Patrono et al. show that DNA damage responses may increase breast cancer risk and radiosensitivity amongst these patients. Aberrant DNA repair BPs is associated with malignancy in breast cancer patients [9]. Similarly, the process of epithelial-mesenchymal transition (EMT) is tightly linked to invasion and metastasis in BLCA [10].

Herein, we aimed to establish a scoring model using biological process-related genes (BPRG) for prognostic prediction and guidance of clinical treatment for BLCA. First, in the TCGA-BLCA cohort, we calculated the expression of 7658 gene sets related to BPs by GSVA and divided BLCA patients into three clusters. Different clusters showed differential prognoses and immune infiltration levels. Subsequently, key genes related to BPs were selected, and a BPRG score model was constructed by the LASSO-Cox method. After classification, the model was found to be closely linked to immune responses and chemotherapy. In conclusion, our results demonstrated the potential relationship of BPRG with prognosis, immune microenvironment, immunotherapy, and chemotherapy in patients with BLCA might be beneficial for developing personalized therapy and assessment strategies.

## 2. Materials and Methods

### 2.1. Raw Data Extraction and Processing

The clinical characteristics and transcriptome of BLCA patients were obtained from the GEO and The Cancer Genome Atlas (TCGA) databases, respectively (GSE13507 and TCGA-BLCA) [11]. The RNAseq and somatic mutations data were downloaded in the FPKM format from TCGA. The average value was taken when multiple Ensembl IDs were mapped to the same gene. All sequencing data downloaded from the GEO database were processed by log quadratic transformation, background adjustment, and normalization. Finally, the data of 565 BLCA patients were retained for further analysis after excluding those without survival information.

### 2.2. Gene Set Variation Analysis (GSVA)

GSVA is a nonparametric unsupervised method [12] to assess the enrichment in sequenced gene sets. The gene expression matrix was transformed into an expression matrix of gene sets to evaluate the differences in enrichment among samples. Herein, the “GSVA” R package and gene sets of BPs in the MSigDB database were employed.

### 2.3. Tumor Subtype Determination

Based on the expression profile of the BP gene sets, consensus cluster analysis was conducted using the “CancerSubtypes” R package to identify the characteristics of different gene sets [13] and classify BLCA patients. Kaplan-Meier analysis was performed to compare differences in the overall survival of patients among clusters. Pearson's chi-squared test was employed to analyze the correlation between clinical factors and clusters; the results were visualized using the “Complex Heatmap” package.

### 2.4. Screening Prognosis-Related Gene Set

The “Limma” R package was used to screen the differential gene sets among the three clusters [14]. Those relevant to survival were selected by univariate Cox and KM survival analyses (a total of 66). Five prognosis-related gene sets were screened by LASSO regression using the “glmnet” package in R [15]. The derived coefficients were utilized to obtain the risk score, and the patients were classified into high- and low-risk groups using the median value of the risk coefficient. The differences in OS between the groups were evaluated by the KM survival curve, and significance was assessed using the log-rank test. The same score calculations, cut-off values, and analysis methods were used to verify the GSE13507 cohort.

### 2.5. Construction of the Predictive Model

112 genes were extracted from the prognosis-related gene sets, and 27 key survival-related genes were screened by univariate Cox and KM survival analyses. All BLCA patients were randomly classified into test (*n* = 282) and training (*n* = 283) groups in the ratio of 1 : 1, following which the Lasso-Cox regression algorithm was executed. The “glmnet” R package was used to reduce the risk of overfitting, and the model was established by 10x cross-validation. Key genes were selected following the multivariate Cox analysis, and BPRG scores were established (BPRG score = *Σ* (Expi ^∗^ coefi)), where coefi and Expi correspondingly indicate each gene's coefficient and expression. The patients were classified into low and high-BPRG score groups based on the median score. The KM survival analysis was performed, and a receiver operating characteristic (ROC) curve was plotted; the findings were verified in the validation set.

### 2.6. Establishment and Verification of the Nomograph Scoring System

Clinical characteristics and BPRG score were utilized to construct the predictive nomogram using the “rms” R package. In the nomograph scoring system, each variable for each sample was matched to a score, and the total score was calculated by adding the addition of the scores of all variables. Nomograms were evaluated using time-dependent ROC curves for 1-, 3-, and 5-year survival. The calibration chart for the nomogram was used to describe the consistency between the predicted 1-, 3-, and 5-year survival events and the obtained results.

### 2.7. Assessment of Immune Status between Different Groups

The ESTIMATE algorithm was used to quantify the abundances of 22 immune cell infiltrates among the three clusters and between the high- and low-BPRG score groups to assess the proportion of tumor-infiltrating immune cells (TIICs) [16, 17]. The correlation of immune functions and interstitial scores with the TME in different groups was compared. Moreover, the benefits of immunotherapy between the high- and low-BPRG score groups were assessed by using the tumor immune dysfunction and exclusion (TIDE) algorithm [18]. The TIDE score was calculated using the TIDE website after normalizing the expression data.

### 2.8. Gene Mutation and Drug Sensitivity Analyses

The mutation annotation format (MAF) in the TCGA database was generated using the “maftools” R package to determine differences in somatic mutations among BLCA patients between the high- and low- BPRG score groups [19]. We also calculated the TMB score for each BLCA patient in both groups, visualized the results using KM curves, and examined the significance of log-rank tests. The R package, pRRophetic, was used to calculate the half-inhibitory concentration (IC50) of commonly used chemotherapeutic drugs in the treatment of BLCA. The differences in their efficacies were determined between the groups [20].

### 2.9. Statistical Analysis

Independent *t*-tests were employed to compare the continuous variables between the two groups. The *x*^2^ test was used for analyzing classified data. TIDE scores between the groups were compared by the Wilcoxon test. KM survival curves were plotted following univariate survival analysis, and the log-rank test was employed to test the significance of the results. The Cox regression model was employed for univariate and multivariate survival analyses. Two-tailed *P* < 0.05 was regarded as statistically significant. All statistical data were analyzed using the packages in R (https://www.r-project).

## 3. Results

### 3.1. Changes in BP Gene Sets between BLCA and Adjacent Normal Tissues

**Figure 1:**
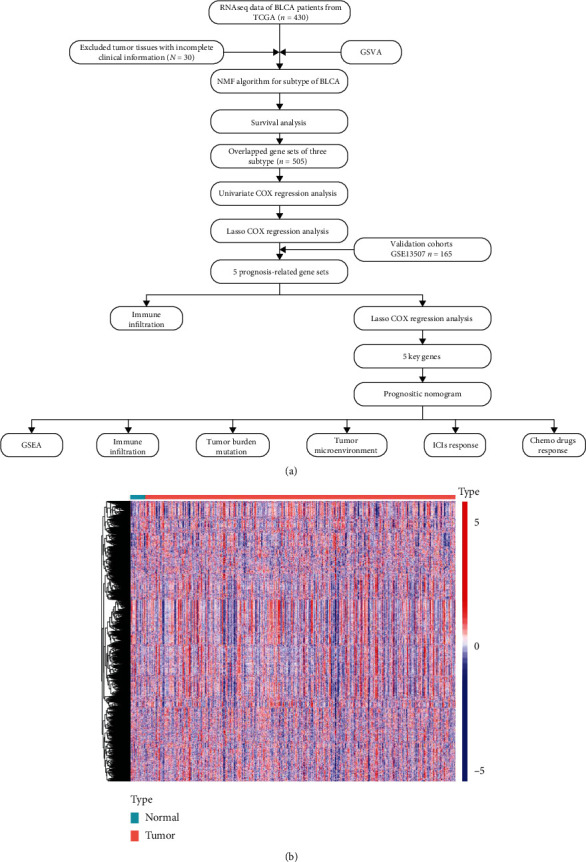
The flowchart of the study design and the heat map of TCGA data. (a) Flowchart of the critical steps in the study. (b) Enrichment score for gene sets of biological processes between cancers and paracancerous tissues in TCGA-BLCA.

To interpret the changes in the enrichment of BP gene sets between BLCA and adjacent normal tissues, first, we downloaded the BP gene set (c5.go.bp.v7.5.1.symbols, including 7658 gene sets) from the GSEA database, following which the BLCA transcriptome and clinical data from TCGA and GEO databases were extracted. TCGA cohort comprised 411 tumor tissues and 19 adjacent normal tissues, while GSE13507 included 165 tumor tissues and 58 adjacent tissues. The flow of this study and the results of the GSVA of the TCGA-BLCA cohort are shown in Figures 1(a) and 1(b).

### 3.2. Determination of BLCA Clusters and their Correlation with Clinical Features

**Figure 2:**
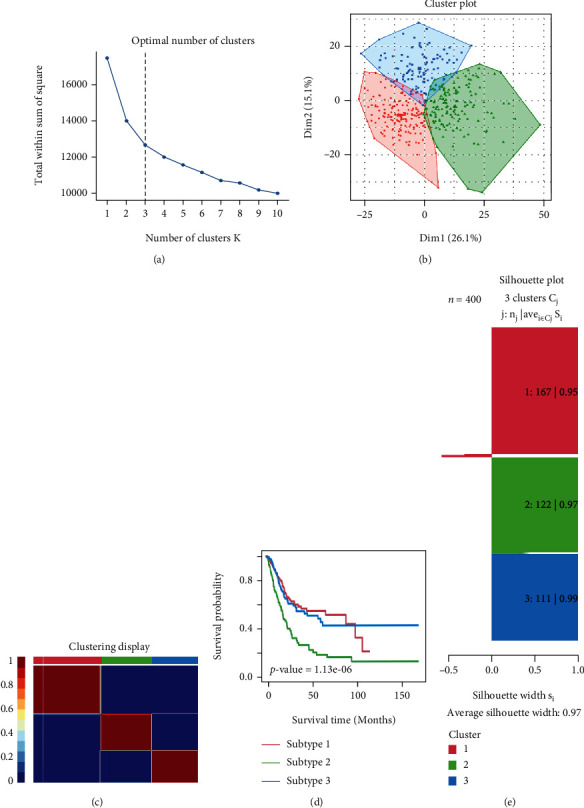
Classification of TCGA-BLCA cohort based on the biological processes enrichment score. (a) BLCA patients were grouped into three clusters according to the consensus clustering matrix for *K* = 3. (b) The PCA scatter plot indicating that three clusters were well distinguished. (c) NMF matrix heat map of each cluster. (d) Kaplan-Meier curves showing three gene clusters that are significantly related to overall survival in BLCA patients (log-rank, *p* < 0.001). (e) Identification of clustering value using silhouette width plots which shows a good accuracy.

TCGA-BLCA was subtyped using the “CancerSubtype” package in R software. The NMF algorithm yielded *K* = 3 as the optimal cluster number, that is, the cohort could be divided into three clusters according to the GSVA-related score of each sample (Figures 2(a)–2(c)). A total of 30 patients were excluded due to the lack of clinical data. In the follow-up study, prognostic differences across the three clusters were analyzed using clinical data (Figure 2(d)). In particular, C1 showed a significantly good prognosis, while C2 exhibited the worst prognosis. The contour width in the figure was 0.97 (Figure 2(e)), which indicated a good match between the BLCA samples and the corresponding clusters.

**Figure 3:**
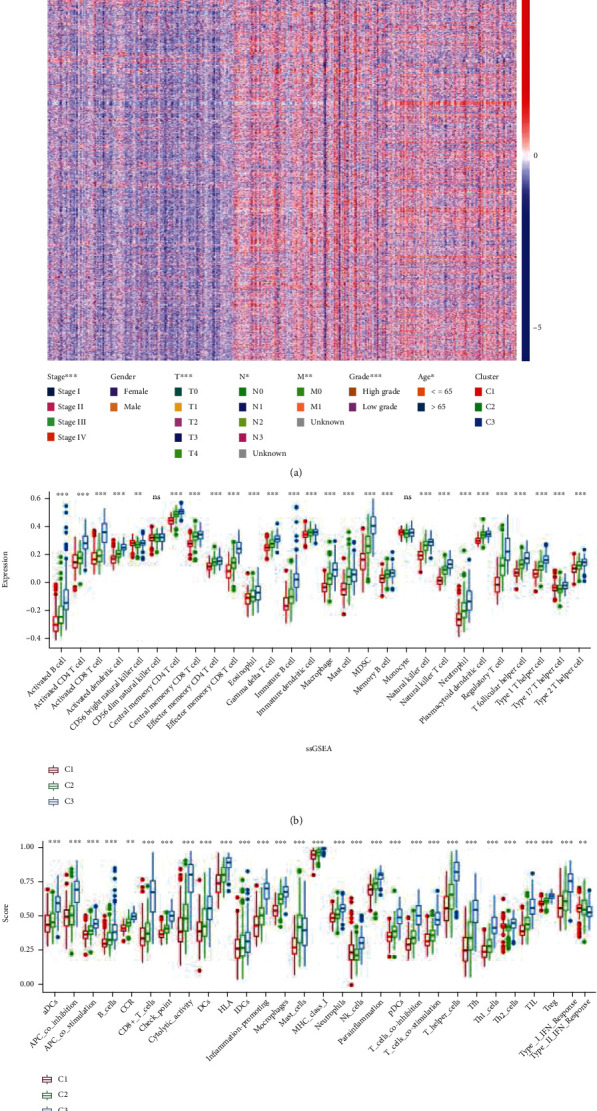
Clinicopathologic and immune infiltration features of 3 clusters in BLCA. (a) Heat map revealing the clinicopathologic features of BLCA patients among 3 clusters. (b) Infiltration of 22 TIICs in 3 clusters. (c) Immune-related functions in 3 clusters. ns: not significant; ^∗^*p* < 0.05; ^∗∗^*p* < 0.01; ^∗∗∗^*p* < 0.001.

Subsequently, we analyzed the clinicopathological differences between three clusters (Figure 3(a)) and found that TNM stage, pathological stage, and histological grade varied in different clusters. In general, as compared to C2 and C3 patients, C1 patients were in the good to medium clinical stage, pathological grade, and TNM stage, and these differences were statistically significant. We further analyzed the differences in TIICs and immune function among different clusters. Twenty-two immune cell types and immune functions had the highest scores in type C3, while the lowest scores were obtained in C1 (Figures 3(b), 3(c)).

### 3.3. Screening Prognostic Gene Sets

**Figure 4:**
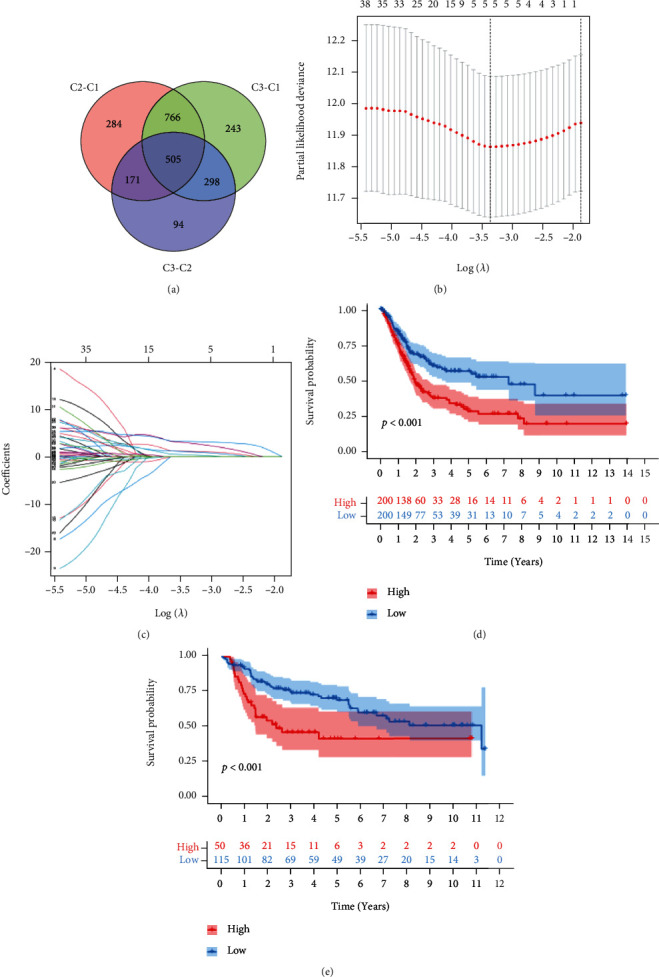
Construction and evaluation of the prognosis-related gene sets based on BP. (a) The common gene sets among differential gene sets between three clusters. (b, c) LASSO regression identified 5 prognosis-related gene sets. (d, e) KM curves of overall survival across different risk groups in the training set and validation set (log-rank, *p* < 0.001).

We calculated the differential gene sets between the three clusters (adj. *p* < 0.05 and |fold change| > 1.69), then took their intersection and combined the gene sets with the clinical data for univariate Cox regression and KM survival analyses. 112 differential gene sets were obtained. Next, these gene sets were analyzed by LASSO regression and cross-validated (Figures 4(a)–4(c)). Finally, 5 target gene sets were determined according to the risk coefficients as follows: regulation of neuron projection regeneration, regulation of cardiocyte differentiation, GOBP convergent extension, regulation of cell fate specification, and relaxation of cardiac muscle. According to the risk score, TCGA-BLCA patients were classified into two groups for survival analysis (Figure 4(d)). The OS survival rate of patients in the low-risk group was higher (*p* < 0.001). The above risk score calculation formula and cut-off value were utilized in GSE13507 to verify the risk score's stability and accuracy, which illustrated that the screening method was meaningful. (Figure 4(e)).

### 3.4. Construction of the Prognostic Model Using Key Genes

**Figure 5:**
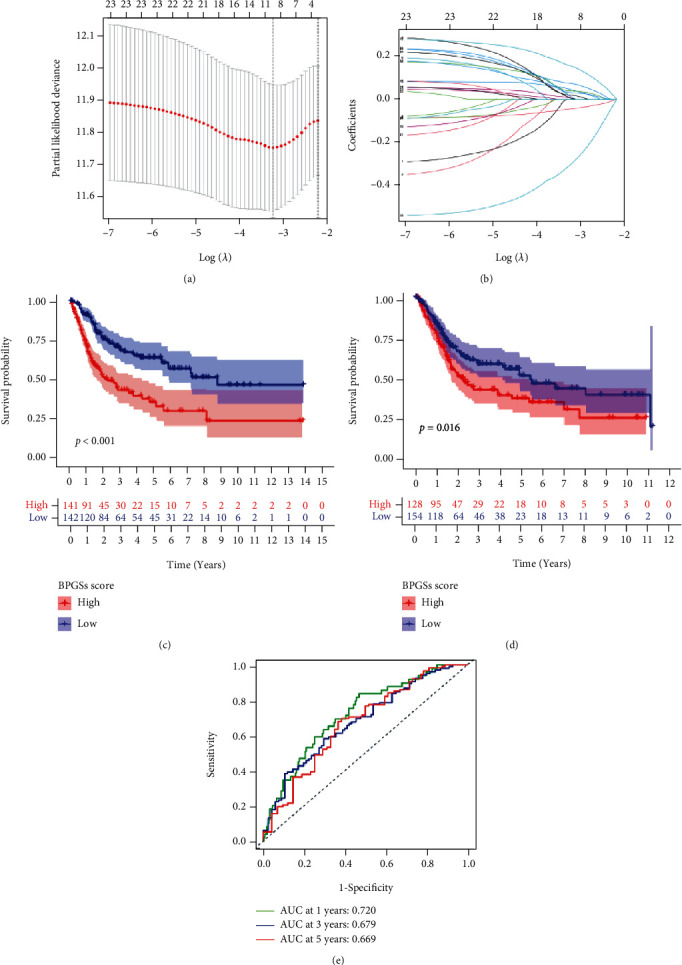
BPRG score and prognosis analysis are constructed based on the training and test cohort. (a, b) Screening the most representative genes by LASSO-COX analysis. (c, d) Kaplan-Meier estimate of the overall survival in the training and test cohorts, divided based on the BPRG score (log-rank, *p* < 0.05). (e) Time-dependent ROC curves of BPRG score.

A total of 112 genes were extracted from the target 5 gene set. After survival and LASSO regression analyses and cross-validation, 5 key genes (PTN, SPP1, KREMEN1, EGFR, and LBX2) were obtained (Figure 5(a–d)). The accuracy of the ROC curve for prognostic prediction was verified. The AUC of 1-, 3-, and 5-year OS survival rates was 0.720, 0.679 and 0.669, respectively (Figure 5(e)).

### 3.5. Establishing the Nomogram

**Figure 6:**
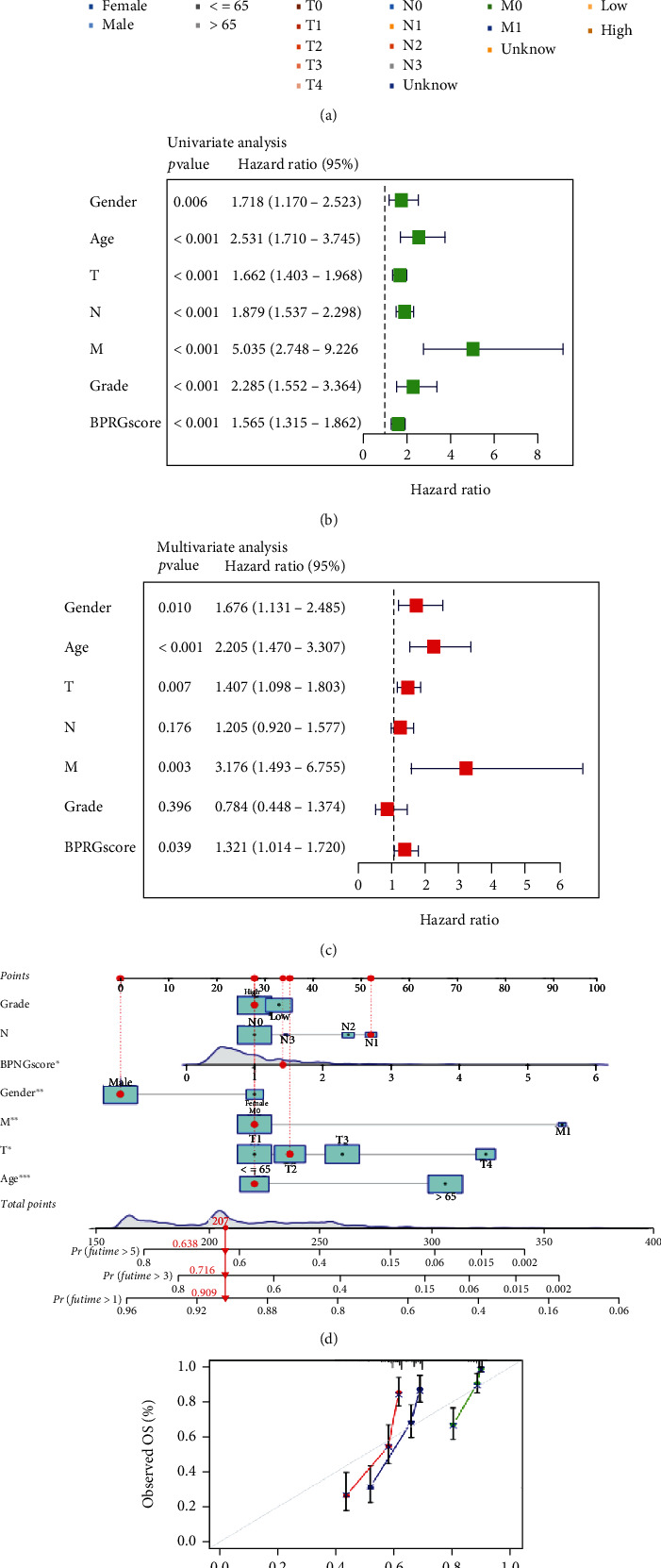
Clinical relevance and nomogram model based on BPRG score. (a) Clinical features for the high- and low-BPRG score groups. (b, c) Forest plot based on univariate and multivariate Cox regression analyses of BPRG score and clinical characteristics. The squares on the transverse lines indicate the HR and the blue transverse lines indicate the 95% CI. (d) Nomograms for predicting the probability of OS based on BPRG score and clinical variables. (e) Calibration plots for validation of the nomogram in 1, 3, and 5-years. ^∗^*p* < 0.05; ^∗∗^*p* < 0.01; ^∗∗∗^*p* < 0.001.

We compared the differences in clinical characteristics between the low- and high-BPRG score BLCA patients to further supplement the clinical applicability of the prognosis model (Figure 6(a)). The results suggested that patients with advanced TNM stage, pathological stage, histological grade, and older age were more frequent in the high-BPRG score group. Univariate Cox regression analysis indicated that gender (*p* < 0.006), age (*p* < 0.001), TNM stage (*p* < 0.001), grade (*p* < 0.001), and BPRG score (*p* < 0.001) were factors for adverse OS. After multivariate Cox regression, the BPRG score was confirmed as an independent prognostic factor (Figures 6(b) and 6(c)). To facilitate the clinical application of the prognostic prediction model, an individualized prediction model was constructed, including age, TNM stage, and pathological grade, and correction curves for 1-, 3-, and 5 years were plotted for 328 patients. Each variable was standardized with a score between 0 and 100; these scores were added to obtain a total score for each BLCA patient to predict the probability of survival at one, three, and five years. The C-index of this nomogram reached 0.742 (95% CI: 0.702–0.781). Thus, the prognosis of TCGA-BLCA patients predicted by the nomogram model was in agreement with the actual prognosis (Figures 6(d) and 6(e)).

### 3.6. Differences in Molecular Characteristics between High- and Low-BPRG Groups

**Figure 7:**
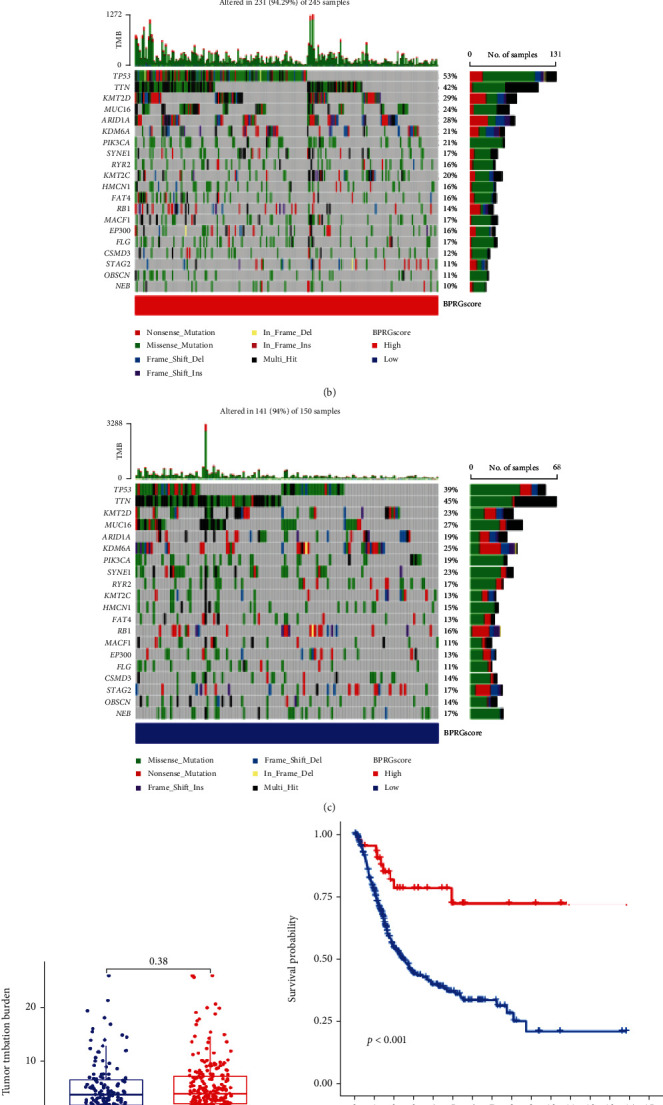
Molecular characteristics of the high- and low-BPRG score groups. (a) The significantly enriched GO and KEGG terms in high-BPRG score group. (b, c) Waterfall plot showing the top 20 mutated genes in high-BPRG score group and low-BPRG score group. (d) Differential analysis of TMB between the two BPRG score groups. (e) Overall survival analysis between different BPRG score groups. (f) Overall survival curves for BLCA patients were classified based on the BPRG score and TMB (log-rank, *p* < 0.001).

GSEA was used to identify enriched gene sets among different groups. Interestingly, activation of immune response, cell adhesion mediated by integrin, chemokine signaling pathway, cytokine receptor interaction, and other pathways related to immune response were enriched in the high-BPRG score group (Figure 7(a)).

Next, gene mutations were analyzed to understand the molecular differences between the two groups. The waterfall diagram shows the top 20 mutated genes in the high score group and the low score group. TP53 exhibited a high mutation frequency in both groups, especially in the high score group (53%) (Figures 7(b) and 7(c)). Although no significant differences were found between the two groups, owing to the fundamental clinical prognostic value of TMB, the TMB of each BLCA patient was calculated and classified into a low TMB group or high TMB group with the optimal cut-off value of 10.158. The survival time of patients with high TMB was significantly longer than that of patients with low TMB. Given the predictive value of TMB and prognostic score for BLCA, we conducted a subgroup survival analysis using the TMB. The overall survival rate was the lowest in the high score and low TMB group (Figures 7(d–f)).

### 3.7. Evaluation of Immune-Related Characteristics

**Figure 8:**
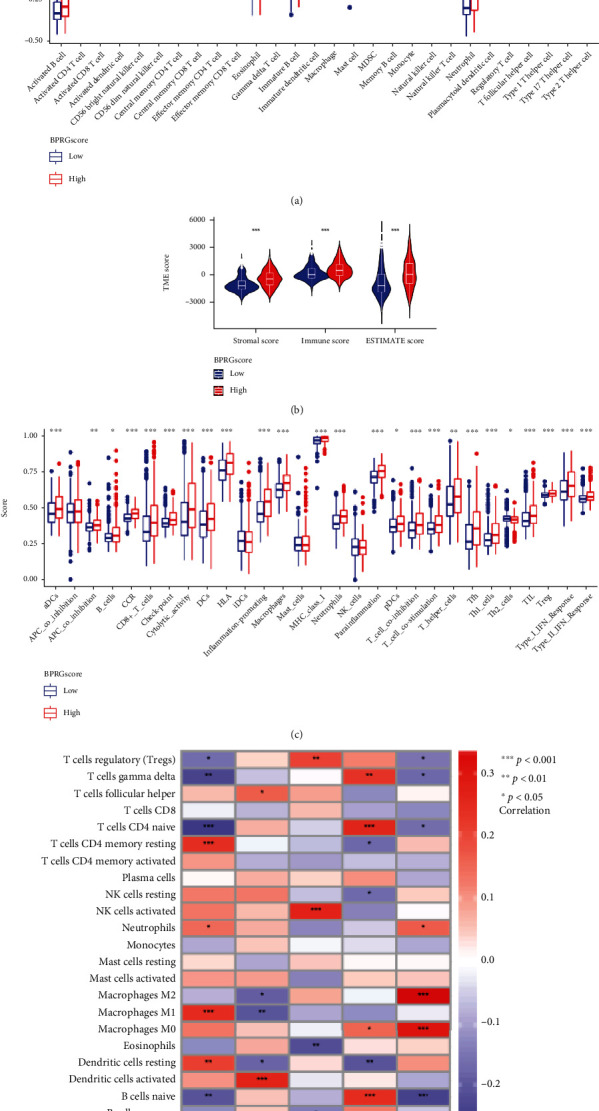
The landscape of the TME between different BPRG score groups. (a) The proportions of immune cell infiltration cells in different BPRG score groups. (b) Violin plot of immune score and stromal score from ESTIMATE. (c) Immune-related functions in different BPRG score groups. (d) Correlation matrix of 5 key genes and immune cell. ^∗^*p* < 0.05; ^∗∗^*p* < 0.01; ^∗∗∗^*p* < 0.001.

Since the immune-related pathways were mainly enriched in the high-BPRG score group, the ESTIMATE and ssGSEA scores were used to compare immune cell infiltration between the two groups. Most immune cells, including memory CD4 and CD8T cells, macrophages, activated CD4 and CD8T cells, and resting natural killer (NK) cells, were abundant in the high score group, while CD56 bright natural killer cells and monocytes were abundant in the low score group (Figure 8(a)). In addition, the differences in immune functions between the two groups were calculated (Figure 8(b)). These results were similar to those of immune infiltration levels. In addition, the ESTIMATE algorithm was employed to calculate stromal and immune scores for each patient; significant differences were obtained between the two groups (*P* < 0.001) (Figure 8(c)). We also analyzed the correlation between 5 key genes and immune cells. (Figure 8(d)).

### 3.8. Correlation between Immunotherapy and Chemotherapy in Different Groups

**Figure 9:**
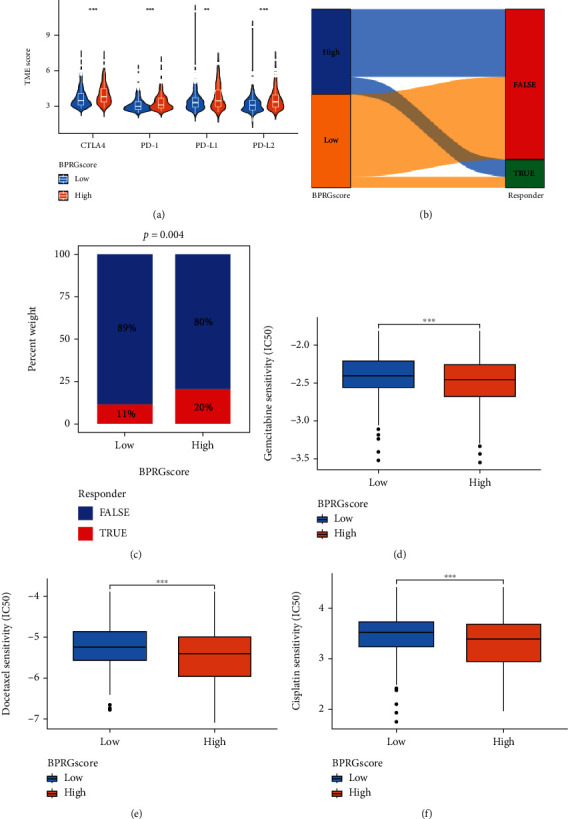
Evaluation of the influence of BPRG score on immunotherapeutic and chemotherapeutics efficacies for BLCA patients. (a) The expression of immune checkpoints in different BPRG score groups. (b) Sankey chart displays the distribution of responder and BPRG score. (c) Comparation of immunotherapy responder among two BPRG score groups. (d–f) Drug sensitivity analysis for gemcitabine, docetaxel, and cisplatin in BLCA patients belonging to high- and low-BPRG score groups. ^∗^*p* < 0.05; ^∗∗^*p* < 0.01; ^∗∗∗^*p* < 0.001.

Subsequently, to examine the role of different scores in antitumor therapy patients with BLCA, we evaluated the correlation between the two groups of patients with immunotherapy and chemotherapy. First, the expression of immune checkpoint-related genes between the two groups was compared (Figure 9(a)), whereby the expression in the high score groups was generally high. The TIDE algorithm was analyzed to predict the immunotherapy responders in BLCA patients. The results showed that the percentage of immunotherapy responders was more efficient in the high-BPRG score group (20%) compared with the low score group (11%) (chi-squared test, *P* = 0.004; Figures 9(b) and 9(c)), which was consistent with the results of immune checkpoint-related gene expression. Since BLCA patients are sensitive to chemotherapy, the IC50 value was measured for each BLCA patient according to the prophetic algorithm. The IC50 of gemcitabine (*P* = 0.0008), docetaxel (*P* = 6.1e − 05), and cisplatin (P = 0.0004) decreased significantly (Figures 9(d–f)). These findings indicated that BLCA patients in the low score group were more sensitive to chemotherapy.

## 4. Discussion

The development and progression of tumors are extremely complex and can be attributed to several genetic abnormalities rather than just one or a few genes. Therefore, gene set-based analysis is attracting increasing attention. Lymphatic metastasis or muscle invasion of tumor cells can affect the treatment outcomes in BLCA and lead to a poor prognosis [21]. In recent years, with further understanding of the relationship of the immune system with the development and occurrence of tumors, immunotherapy is a sought-after treatment strategy for BLCA [22]. However, because BLCA is a highly heterogeneous tumor, these patients usually show different prognoses. Therefore, several studies have focused on identifying new biomarkers to guide individual immunotherapy and predict prognosis.

As machine learning technology proved good accuracy in classification and identification [23, 24]. Herein, after GSVA for the BP gene sets, we classified the patients in the TCGA-BLCA cohort according to the levels of gene set expression. As shown in Figure 2, the classification based on the result of GSVA was relatively accurate, and the average contour area reached 0.97. In our study, three clusters showed significant differences in prognosis, immune infiltration levels, and functions. Due to this method being based on gene sets expression, although the accuracy was high, the applicability may not be substantial. Thus, after extracting the target genes from different gene sets, LASSO-Cox regression was performed twice to analyze the dimensionally reduced target genes, and finally, 5 key genes were obtained (PTN, SPP1, KREMEN1, EGFR, and LBX2). According to the calculated cut-off value, the patients were classified into low- and high-BPRG score groups. In the test group, survival time in the low score group was longer. Similar results were obtained in the validation group. The higher the score, the worse the clinical features and the shorter the expected survival time. Thus, we constructed the nomogram and plotted the correction curve, which could predict the patients' OS more accurately and was more conducive to clinical application.

Some previous studies have reported these 5 key genes, while others have also shown their close relationship with cancer. Pleiotroin (PTN) is a protein-coding gene. The protein encoded by this gene is a secretory heparin-binding growth factor. PTN promotes nerve invasion in pancreatic cancer [25]. Overexpression of SPP1 is related to a poor prognosis of melanoma. Silencing SPP1 inhibits melanoma cells' migration, invasion, and proliferation [26]. Somatic mutations in KREMEN1 found in human cancer can affect proapoptotic activity and support tumor suppressive functions [27]. EGFR is often expressed at high levels in different cancers, and its expression positively correlates with tumor progression and poor prognosis [28]. EGFR is also activated in basal cells, including those in BLCA; its overexpression is also associated with poor prognosis [29]. LBX2 serves carcinogenic functions in LUAD and is involved in tumor migration, proliferation, and invasion throughout EMT processes [30].

Interestingly, after GSEA of key genes in the high score group, the pathways related to cancer promotion and immune activation were enriched in the high score group, including activation of immune response, cell adhesion mediated by integrin, chemokine signaling pathway, and cytokine receptor interaction. Integrins, chemokines, and cytokines are also closely related to cancer. According to Cooper and Giancotti, integrin signaling can drive several tumor cell functions, including initiation, metastasis reactivation, and epithelial plasticity, along with resistance to oncogene and targeted immune therapies [31]. Bagati et al. show that integrin *α*v*β*6 can promote immune escape in breast cancer [32]. In BLCA, Liu et al. show that integrin *β* can promote drug resistance and growth in these cells through the Y-box binding protein 1-dependent signaling pathway [33]. Chemokines can participate in the generation and recruitment of immune cells, which help in forming a microenvironment that promotes tumor progression [34]. Considering that the key genes were closely related to immune functions, immune responses are important for the occurrence and treatment of BLCA [35]. We then compared the differences in TIICs, immune-related functions, interstitial cell infiltration levels, immune checkpoints, and immunotherapy between the groups. In the high score group, the abundances of immune cells including CD8 cells and macrophages were high, and the related immune functions were enriched. Moreover, the interstitial score of the high score group was also higher. Stromal and immune cells are the main components of TME. High immune and stromal scores are associated with poor prognosis [36]. This suggested that the high evaluation group may show a poor prognosis.

The patients in the high score group also showed increased expression of immune checkpoint-related genes, including PD1, PDL1, and CTLA4 which suggested that the high score group may benefit more from immunotherapy [37]. The immune checkpoints serve regulatory functions in immune responses by inhibiting the initiation of immune cell responses and immune monitoring [38, 39]. TIDE is a computational framework that simulates the two main mechanisms of tumor immune escape. It predicts the results of immunotherapy [18]. Subsequently, we verified our hypothesis using the TIDE algorithm, and the results were consistent with previous findings for immune cell infiltration and expression of immune checkpoint-related genes. Unexpectedly, the high rating group showed a lower survival rate. We further evaluated the differences in somatic mutation data between the two groups. The three genes with the highest mutation rates were TTN, KMT2D, and TP53 in BLCA, similar to Song et al. [40] Our calculations showed that the high TMB group had a longer survival time, which is also in line with the conclusion of Samstein et al. [41]. Moreover, high TMB cannot predict the immune checkpoint blockade responses in all cancer types [42]. A high level of immune checkpoint gene expression may stimulate the microenvironment of immunosuppression and lead to the immune escape of tumor cells [43]. Therefore, tumor immunity is a complex and detailed mechanism, and one-sided predictions cannot be made using unilateral factors. Therefore, we speculated that the high score group did not show a good prognosis due to the activated immune pathway, and high expression of immune cells were eliminated by the presence of other cells in the immune microenvironment, such as stromal cells. Simultaneously, high expression of immune checkpoints also exerts synergistic regulatory effects. Next, the TCIA database was used for analyzing the chemosensitivity between the two groups. Overall, the chemosensitivity in the low score group was significantly higher than that in the high score group, especially in treatment using gemcitabine, docetaxel, and cisplatin. This finding suggested that chemotherapeutic drugs may be more effective in the low score group, and thus, the score is a potential predictor of chemotherapeutic benefits in BLCA patients.

However, there are some limitations to this study. First, this was a retrospective study based on publicly available databases. There may be some biases in patient selection and relevant clinical information. Further research is needed to verify our conclusions. Second, the established model did not have a good predictive value for some immunotherapy patients, which may be related to the highly heterogeneous feature of BLCA. Therefore, more extensive research is needed, including single-cell sequencing analysis to more accurately explain the specific changes in the TME. Finally, these findings need to be verified through in vivo and in vitro experiments in the future.

## 5. Conclusions

In conclusion, we systematically analyzed the relevant information on key genes screened based on BPs and found significant differences in clinical characteristics, immune microenvironment, immunotherapy, and chemotherapy between the low and high score patients. The findings demonstrated the clinical significance of comprehensively evaluating the BPRG score for each BLCA patient, which is expected to facilitate the formulation of individual therapy strategies by oncologists.

## Data Availability

The data used to support the findings of this study are available from the corresponding author upon request.
